# Breaking the Chain of Infection: A Systematic Review of Environmental Decontamination of *Candidozyma auris* (2017–2025)

**DOI:** 10.3390/jof12020131

**Published:** 2026-02-11

**Authors:** Aristotelis Papadimitriou, Lida-Paraskevi Drosopoulou, Maria Tseroni, Flora V. Kontopidou, Athanasios Tsakris, Georgia Vrioni

**Affiliations:** 1 General Oncology Hospital of Athens “Saint Savvas”, 11522 Athens, Greece; lpdrosopoulou@agsavvas-hosp.gr; 2 Department of Microbiology, Medical School, National and Kapodistrian University of Athens, 11527 Athens, Greece; atsakris@med.uoa.gr; 3 Nursing Department, National and Kapodistrian University of Athens, 11527 Athens, Greece; mtseroni@nurs.uoa.gr; 4 Mitera General Hospital Athens, 15123 Athens, Greece; fkontopidou@hygeia.gr

**Keywords:** infection control, *Candidozyma auris*, disinfection, biofilm

## Abstract

*Candidozyma auris* is an emerging multidrug-resistant yeast that readily contaminates healthcare environments, persisting on dry surfaces and enabling transmission and difficult-to-control outbreaks. A systematic review of environmental hygiene interventions targeting *C. auris* was conducted, focusing on efficacy against planktonic cells and surface-associated biofilms (including dry-surface biofilms, DSB where available). PubMed and Scopus were searched for English-language records published from 1 January 2017 to 30 September 2025, and study selection followed PRISMA 2020. Thirty-six studies from nine countries met the inclusion criteria. These were predominantly laboratory efficacy evaluations using carrier/suspension or quantitative surface methods reporting log_10_ Colony Forming Unit (CFU) reductions; only seven studies assessed biofilm-associated *C. auris*. Across clades I–IV, chlorine-based disinfectants and oxidizing chemistries (hydrogen peroxide/peracetic acid formulations) most consistently achieved high-level reductions (often ≥ 5 log_10_ CFU) under label-relevant conditions. In contrast, products containing only quaternary ammonium compounds (QACs) frequently underperformed and demonstrated greater variability. No-touch methods, particularly 254 nm ultraviolet-C light (UV-C), provided meaningful adjunctive reductions, but were highly dependent on dose delivery and geometry, and evidence for ozone-based approaches was mixed. Limited data on *C. auris* DSBs suggest planktonic testing may overestimate real-world conditions and underscore the importance of endpoints, such as transfer prevention and regrowth suppression.

## 1. Introduction

Healthcare-Associated Infections (HAIs) remain a major, preventable cause of morbidity and mortality, prolonging hospitalization, straining limited resources, and increasing costs. At the same time, antimicrobial resistance (AMR) is expected to drive substantial global impacts on mortality and healthcare expenditure [1]. Strengthening infection prevention and control (IPC) is therefore essential, and a key IPC pillar is healthcare environmental hygiene (HEH), or the microbiological dimension of hospital cleaning and disinfection. The healthcare environment is a recognized reservoir for pathogens, including multidrug-resistant organisms (MDROs). Improved cleaning and disinfection of shared equipment can reduce HAIs, and stronger environmental hygiene is associated with lower environmental bioburden and reduced patient colonization [2,3].

Although MDR bacteria often dominate IPC priorities, fungal HAIs are increasingly recognized, including infections caused by drug-resistant fungi. *Candidozyma auris* (formerly *Candida auris*) is an emerging multidrug-resistant yeast associated with healthcare transmission and difficult-to-control outbreaks [4,5]. Since its first description in 2009, *C. auris* has been reported across all inhabited continents and in more than 40 countries, with a marked rise in cases during the COVID-19 pandemic [6]. The fungus rapidly reached endemicity in many countries in the world, mainly in Asia. Data from the European Center for Disease Prevention and Control (ECDC) indicate that Greece, Italy, Romania, and Spain reached regional endemicity rapidly after first detection, underscoring how quickly *C. auris* can spread in hospitals. Conversely, there are also examples where transmission was prevented or contained [7,8].

Its public health significance is underscored by its inclusion in the World Health Organization’s fungal priority pathogens list since 2022 and by its designation by the U.S. Centers for Disease Control and Prevention (CDC) as an urgent threat; it is the first fungus to receive this designation [9,10].

*C. auris* has several “superbug-like” traits that collectively support healthcare persistence and transmission. It often exhibits intrinsic or acquired resistance to azoles, and resistance to amphotericin B and echinocandins is increasingly reported. A defining epidemiologic feature is persistent skin colonization paired with exceptional adaptation to the healthcare environment. Infected patients can shed organisms onto high-touch surfaces and shared equipment, and the yeast can persist on surfaces for prolonged periods, enabling indirect transmission and recontamination of cleaned areas [4,11].

Thermotolerance has been hypothesized as an adaptation to rising environmental temperatures, potentially facilitating the ability to cross the mammalian thermal barrier. Accordingly, *C. auris* has been discussed as a possible example of pathogen emergence influenced, at least in part, by climate change. In parallel, agricultural azole use has been proposed as a driver contributing to widespread azole resistance [12,13].

On human skin, *C. auris* can form multilayer biofilms, reside in hair follicles, and may penetrate deeper layers. Skin colonization has been linked to Surface Colonization Factor (SCF1), a *C. auris*-specific adhesin that supports surface association and biofilm formation, contributes to environmental persistence and colonization, enhances virulence in invasive infection, and promotes adherence to indwelling devices such as catheters [14]. Patients may remain infected for prolonged periods, yet standardized decolonization protocols are not established despite reports describing topical antiseptics (most commonly chlorhexidine) [15]. Experimental work using synthetic sweat suggests *C. auris* can form dense biofilms, tolerate evaporation, and resist dehydration. These features likely support persistence on skin and inanimate surfaces and facilitate spread in healthcare settings [16].

In a large U.S. case-based surveillance study (2016–2023), 6.9% of screened-positive patients progressed to clinical disease (2.8% candidemia) often weeks to months after initial detection. Since skin colonization is a key risk factor for subsequent infection, rigorous IPC—including environmental disinfection and adherence to standard and contact precautions—particularly during outbreaks and in endemic settings, is essential [17].

Environmental contamination is central to *C. auris* transmission. Infected patients can shed the organism into the healthcare environment, where it may persist and contribute to transmission directly or via shared equipment. Data show that the pathogen can remain metabolically active on surfaces for at least four weeks in viable but not culturable state (VBNC), which may hinder detection by standard laboratory methods [18]. During outbreaks, extensive contamination of rooms and equipment is common, and contamination correlates strongly with patient skin burden, consistent with patterns observed for other important pathogens (i.e., carbapenem-resistant *Enterobacterales* and *Clostridioides difficile*) [19]. In an article describing one of the primary outbreaks of *C. auris*, the minimum contact time with a positive patient or contaminated environment required for acquisition was four hours [20].

Successful containment requires bundled IPC measures, including active surveillance and enhanced environmental hygiene, and evidence from settings that prevented spread even under high endemic pressure supports this approach [21]. Current guidance from the CDC, ECDC, United Kingdom Health Security Agency (UKHSA) and expert opinions recommend rigorous environmental disinfection of high-touch areas and shared equipment, often emphasizing hospital-grade disinfectants with demonstrated activity against *C. difficile* spores, as part of broader IPC strategies to minimize cross-transmission [22,23].

Multiple studies have evaluated *C. auris* susceptibility to high- and low-level disinfectants; overall effectiveness depends on correct implementation (i.e., dilution, concentration, contact time, and appropriate pre-cleaning). By contrast, quaternary ammonium compound (QAC)-only products have frequently shown limited activity. In addition, tolerance may be mediated by molecular mechanisms and/or biofilm-associated protection that supports survival and regrowth [24,25,26].

Biofilms are structured microbial communities embedded in extracellular polymeric substances (EPSs) and are associated with enhanced tolerance to antimicrobials and disinfectants. While hydrated (“wet”) biofilms are well studied, environmental dry-surface biofilms (DSBs) have gained attention more recently and appear widespread in healthcare environments. The term DSB was introduced in 2015. They may facilitate long-term survival under dehydrating conditions, resist physical removal and chemical disinfection, and potentially contribute to persistent contamination and transmission, even though direct links to outbreaks are not always fully established [27,28,29].

*Candida* spp. biofilm formation is an important virulence factor, and unlike commensal *Candida* species, *C. auris* spreads efficiently via patients and contaminated fomites, survives on diverse substrates, and can persist despite daily cleaning and disinfection [30]. The biofilm-forming feature contributes to *C. auris* virulence and underpins its ability to cause outbreaks through enhanced environmental persistence [31].

In vitro studies suggest that biofilm formation is a survival mechanism for *C. auris* facilitating its persistence in the hospital environment. Even chlorine-based agents, such as sodium hypochlorite (NaOCl), widely used in healthcare, can show variable activity depending on concentration, exposure time, and strain phenotype (i.e., aggregative versus non-aggregative), although oxidative chlorine-based compounds remain widely regarded as a cornerstone for surface and equipment disinfection due to their broad antimicrobial spectrum [32].

Invasive fungal infections are rising, particularly among critically ill patients, individuals with cancer, and those with severe immunocompromise; however, fungal HAIs have historically received less attention than drug-resistant bacterial infections [33]. The rapid and extensive environmental contamination associated with *C. auris*—often extending beyond the immediate patient surroundings—can drive ongoing transmission, outbreaks, and progression to endemicity, reinforcing the need to evaluate IPC strategies with particular attention to environmental hygiene [34,35].

Despite expanding guidance and a growing research base, uncertainty persists regarding which disinfectant classes and modalities achieve reliable reductions under clinically relevant conditions, including both planktonic contamination and surface-associated biofilm states, such as DSB. A synthesis of available evidence is needed to inform product selection and protocol design, strengthen outbreak response, and define priorities for future experimental and implementation research.

This study aimed to review and synthesize evidence on environmental hygiene interventions targeting *C. auris* in healthcare relevant contexts, including efficacy against planktonic cells and surface-associated biofilm models (especially DSB where available), and to evaluate how study design features (i.e., test method, strain/clade, soil load, and setting) may explain variability in outcomes. By integrating laboratory and field evidence and mapping it onto current guidance, this review aims to clarify what is known, where uncertainties persist, and which research and practice priorities are most likely to reduce environmental reservoirs and interrupt transmission

## 2. Materials and Methods

**Figure 1:**
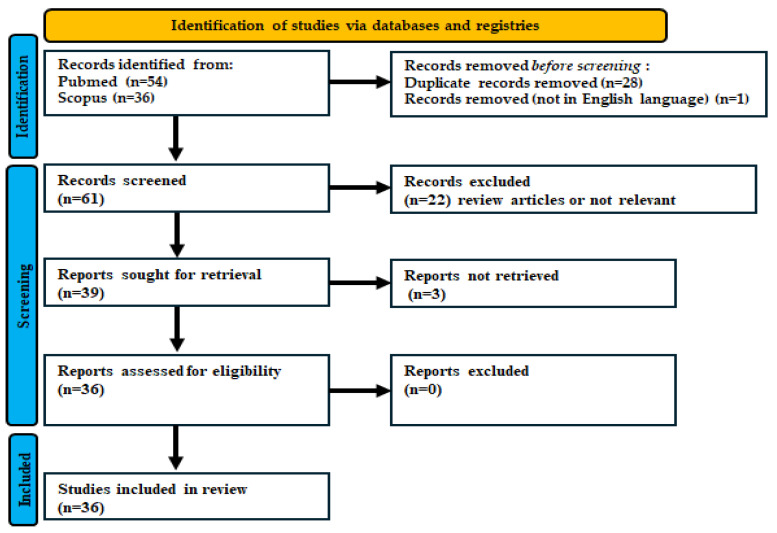
PRISMA 2020 flow diagram of the study selection process.

PubMed and Scopus were investigated for records published between 1 January 2017 and 30 September 2025. The search employed keywords related to *C. auris* and environmental hygiene/disinfection in healthcare settings, using the terms “*Candida auris*”, “disinfection”, “cleaning”, “environmental” (*Candida auris* AND disinfection OR decontamination OR cleaning AND environmental). The PRISMA 2020 guidelines were implemented for the systematic review [36]. The selection process is summarized in the PRISMA flow diagram (Figure 1). The review was planned and reported in accordance with the PRISMA 2020 statement. The protocol was registered in PROSPERO (ID: CRD4202511444474).

Original research articles in English were included in the study that evaluated strategies for decontaminating healthcare environments contaminated with *C. auris*, including chemical disinfectants and non-chemical modalities (i.e., ultraviolet devices) tested on relevant surfaces, equipment, or environmental matrices. Review articles, editorials, conference abstracts without full data, and studies not focused on environmental disinfection of *C. auris* were excluded from the study.

Search results from each database were exported to a shared screening file and duplicates were removed prior to screening. Two reviewers independently (A.P. and L.-P.D.) screened titles and abstracts against the eligibility criteria, followed by full-text assessment of potentially relevant reports. Discrepancies were resolved by discussion and, when needed, by adjudication from a third reviewer (G.V.).

## 3. Results

### 3.1. Study Characteristics and Evidence Base

Across the extracted dataset, 36 studies from nine countries evaluated interventions to inactivate or remove *C. auris* on abiotic materials. Most assessed chemical disinfectants (chlorine-based agents, QACs, hydrogen peroxide, and alcohols), while a smaller subset evaluated non-chemical approaches such as UV radiation. Seven studies assessed biofilm-associated *C. auris*, while the remainder focused on planktonic/vegetative cells or dried inocula on carriers. Isolates from all major clades (I–IV) were represented, although clade coverage varied and many experiments used a single strain per assay.

The assessment of the methodological quality under the JBI Critical Appraisal Checklist and CASP resulted in fourteen studies receiving a score of 10, eleven of 9 and eleven of 8. According to the scores, 25 studies were classified as high quality and the remaining 11 as moderate methodological quality.

The included studies were predominantly laboratory efficacy evaluations (carrier/disk methods, suspension tests, and in vitro biofilm models), with a smaller subset using applied environmental hygiene workflows (i.e., floor cleaning plus adjunct UV). The majority of studies used standardized carrier tests (i.e., stainless steel/polymer carriers, polystyrene plates, or simulated surfaces), reporting outcomes mainly as log_10_ CFU reduction at defined concentrations/wavelengths and contact times. A key finding was the differential efficacy observed between classes of chemical agents, where oxidizing agents (like chlorine) typically achieved higher and more consistent log reductions than non-oxidizing agents (like certain QACs). Several investigations explicitly compared multiple clades (I–IV), demonstrating clade- and strain-dependent variability. Performance varied by formulation and test conditions. Studies were performed across nine countries (Table 1), with the largest number conducted in the USA (*n* = 21) and UK (*n* = 5), followed by South Africa (*n* = 3), Austria (*n* = 2), and single studies from Turkey, China, Canada, Poland, Germany (*n* = 1 each).

For environmental hygiene interventions against *C. auris*, most evidence evaluates chemical disinfectants, with fewer studies assessing no-touch methods (mainly UV-C) or antimicrobial surfaces. Overall, 36 studies (Table 1) examined inactivation or removal from diverse abiotic substrates (i.e., steel/polymer carriers, plastics, glass, and polystyrene plates), and most were laboratory efficacy experiments (carrier/suspension or quantitative surface methods) reporting log_10_ CFU reductions at defined concentrations/wavelengths and contact times. Multiple clades (I–IV) were represented, and several studies reported strain- or clade-dependent variability under specific conditions. Only a minority of studies explicitly evaluated biofilm-associated *C. auris*, including two studies using a DSB model.

### 3.2. Oxidizing Disinfectants

Chlorine-based agents were consistently among the most effective chemistries. In a multi-clade comparative study, sodium hypochlorite (NaOCl) 0.65% (wipe and dilution) achieved >5 log_10_ reduction within 3 min, and NaOCl 0.63% (wipe) achieved >5 log_10_ within 4 min across clades I–IV. Similarly, sodium dichloroisocyanurate (NaDCC) 4306 ppm achieved >5 log_10_ reduction within 4 min across clades [56]. In ASTM quantitative carrier testing, multiple commercial bleach formulations containing 0.39–0.825% NaOCl produced ≥5 log_10_ reductions within 1 min [64]. However, lower chlorine concentrations demonstrated more variable performance. In surface/carrier comparisons, 1000 ppm NaOCl produced approximately 1–3 log_10_ reductions depending on contact time and material, while higher concentrations improved performance [25]. Using EN-standard suspension and carrier methods, chlorine activity against *C. auris* was strongly dependent on concentration, contact time, and test conditions. At 200 ppm, short exposures (1–5 min) were not fungicidal, with meaningful reductions only at longer contact times, whereas 500 ppm achieved stronger effects in suspension testing but showed reduced performance in dirty-condition carrier assays and against some strains, highlighting the impact of organic load and isolate variability on apparent efficacy [41]. A laboratory model using a 12-day DSB model showed that chlorine efficacy differs markedly by growth mode. In planktonic suspension, *C. auris* (six clinical isolates; clades I and III) was highly susceptible to NaOCl 500–1000 ppm (available chlorine) for 1–5 min, achieving ≥6-log_10_ reductions with no CFU detected after neutralization. However, when grown as DSBs over repeated wet/dry cycles, the same NaOCl conditions produced substantially smaller reductions (~2–4 log_10_ reductions) at the highest concentrations, consistent with increased tolerance in mature DSBs (see Section 3.5) [71]. A main disadvantage of sodium hypochlorite is its corrosiveness, thus rendering the agent incompatible with several surfaces in the hospital environment and can be irritating to healthcare personnel and patients [59].

Other oxidizing chemistries also demonstrated high efficacy under recommended concentrations and contact times. In multi-clade testing, a peracetic acid/hydrogen peroxide formulation (PAA 1200 ppm + H_2_O_2_ < 1%) achieved >5 log_10_ within 3 min [56]. Hydrogen peroxide products similarly achieved high-level reductions: in multi-clade comparisons, 0.5% H_2_O_2_ produced ~4.78–5.74 log_10_ reductions (with lower reduction reported for clade IV), while a low-strength H_2_O_2_ concentration (0.07%) produced 4.02 log_10_ for clade II but only 1.48 log_10_ for clade I and was not effective for clades III–IV [50,56]. In ASTM carrier testing, 1.4% H_2_O_2_ and 0.5% H_2_O_2_ products achieved ≥5 log_10_ reductions [64]. In the EPA SOP-MB-35 disk carrier method, peroxide-based products again achieved ≥5 log_10_ reductions at short contact times, supporting oxidizing disinfectants as reliable options under standardized quantitative test conditions [65].

Aerosolized/room decontamination oxidizers were more heterogeneous. A PAA + H_2_O_2_ aerosolized system (22% H_2_O_2_, 4.5% PAA) achieved ≥5 log_10_ reduction on steel carriers and portable equipment within 21 min [38]. In contrast, one silver-stabilized aerosolized hydrogen peroxide approach demonstrated only modest reductions in the reported conditions [46].

### 3.3. Quaternary Ammonium Compounds (QACs)

Across studies, QAC formulations exhibited variability and frequently underperformed compared with chlorine and oxidizers. In multi-clade comparisons, QAC products did not achieve >5 log_10_ reductions across clades at tested contact times [56]. In an EPA MB-35 study, QAC-only products produced low reductions (i.e., ~0.25–1.82 log_10_ at 10 min), whereas QAC + alcohol products achieved ≥5.29 log_10_ reductions at 1–2 min [65]. Notably, multi-clade data suggested that some QAC + alcohol formulations retained clade dependence; one formulation achieved >5 log_10_ for clade II but <5 log_10_ for other clades [56].

### 3.4. Alcohol-Based and Phenolic Disinfectants

Alcohol-based products (i.e., 70% ethanol) showed high reductions in some contexts under clean and dirty conditions [41,52], but outcomes were not always reported as CFU-based log_10_ reductions across all studies. Phenolic formulations performed poorly in multi-clade testing, with no tested strains achieving >5 log_10_ reduction at 10 min under the reported conditions [56]. Non-standard agents such as acetic acid and low concentration alcohol products produced only ~2–3 log_10_ reductions in ASTM carrier testing [61].

### 3.5. Biofilm-Specific Findings

Simultaneous assessment of biofilm versus planktonic/vegetative state phenotypes resulted in biofilms being generally harder to eradicate than planktonic cells, and susceptibility was sometimes strain dependent. In a 24 h biofilm model, several surface disinfectants achieved >5 log_10_ reductions for both planktonic and biofilm conditions, yet hydrogen peroxide performance in biofilms varied by strain (including incomplete biofilm eradication for some conditions) and metabolic assays sometimes indicated residual activity despite large viable-count reductions [69]. Biofilm-disrupting formulations achieved high reductions in both planktonic and biofilm settings (including near-complete multi-log reductions under specified conditions) in one study, supporting the concept that disrupting the extracellular matrix may improve fungicidal outcomes [57].

One study directly quantified a maturity-dependent decline in NaOCl kill in DSBs: for strain NCPF8973, 500 ppm for 1 min decreased from 4.5 log_10_ (cycle 1) to 1.2 log_10_ (cycle 3), and for NCPF8978, 1000 ppm for 5 min decreased from 6.7 log_10_ (cycle 1) to 3.0 log_10_ (cycle 3). This reinforces that biofilm growth on dry surfaces can enable persistence despite chlorine exposure that is otherwise fully effective against planktonic cells. Transcriptomic profiling comparing untreated DSBs vs. planktonic cells identified upregulation of transmembrane transport, including ABC transporters such as CDR genes and iron acquisition pathways in DSBs, suggesting plausible mechanisms contributing to environmental survival and reduced NaOCl susceptibility [71]. A similar study used a 12-day DSB model on stainless steel disks, created through repeated hydration desiccation cycles under a modified ASTM E2967-15 wipe protocol (mechanical wiping with 500 g pressure for 10 s, 2 min contact, followed by Dey-Engley neutralization), quantified log_10_ reduction, transferability after wiping and regrowth delay. In this DSB context, only a limited subset of formulations performed consistently well across endpoints: a 3500 ppm peracetic acid wipe product and a formulated 1000 ppm NaOCl product prevented detectable transfer after wiping, and several chlorin and/peracetic acid products achieved >7 log_10_ reduction in viable cells embedded in DSB. Despite measurable CFU reductions for some agents, the authors reported substantial failure rates across clinically relevant outcomes: 50% of tested products did not meaningfully reduce viability, 58% failed to prevent transferability, and 75% did not delay regrowth, highlighting that log_10_ reduction alone can underestimate residual transmission risk when viable transfer and rebound growth persist [70]. This aligns with broader biofilm focused findings that endpoint choice (viability vs. persistence/transfer/regrowth) can substantially alter the interpretation of “effective” disinfection [69].

### 3.6. No-Touch Methods

Multiple studies supported 254 nm UV-C as an effective adjunct, but efficacy varied by dose delivery, device configuration, distance, and exposure time. A wavelength-sensitivity study also reported ~4.81 log_10_ reduction within 40 s at 267–270 nm and described effective antibiofilm activity [45]. A mobile 254 nm tower produced >3.86 log_10_ reduction (≥99.97%) within 7 min at ~2.4 m distance in a room-sized test chamber [54]. In a floor-tile model, 254 nm UV-C at 91 cm distance, for 10 min reduced *C. auris* to non-detectable levels (reported as >3.85 log_10_ reduction), comparable to high-performing chemical strategies when applied with appropriate contact time and removal steps [63]. Device geometry is important, at the same nominal delivered dose (575 mJ/cm^2^), one 254 nm emitter configuration achieved >6 log_10_, whereas an alternative configuration achieved only ~1.66 log_10_, indicating substantial sensitivity to irradiation uniformity and design [62]. Other UV-C studies demonstrated more modest reductions under certain deployment conditions and strain combinations [40,43]. Far UV-C (222 nm) showed time-dependent effects, with longer exposures producing higher reductions. Reported outcomes ranged from <3 log_10_ (not effective) at 45 min in one experiment to >2 log_10_ at 4 h and >3 log_10_ at 12 h in another setting [39,60,61]. UV-A (365 nm) produced only modest reductions (approximately 0.7 log_10_ after prolonged exposure), suggesting limited utility as a rapid standalone approach [42].

Ozone demonstrated stronger inactivation of planktonic cells than biofilms in a disinfection chamber study. Under the study’s tested conditions, ozone achieved approximately ~3.3 log_10_ reduction in biofilm, whereas UV-C achieved approximately ~7.2 log_10_ reduction in biofilm over comparable exposure duration; planktonic cells reached ~8 log_10_ reductions across modalities. A hybrid strategy combining ozone and UV-C improved biofilm reductions relative to ozone alone [68]. One ozone room-device study also reported moderate multi-log reductions under its specific operating conditions [44].

### 3.7. Emerging and Adjunctive Approaches

Antimicrobial surfaces present as emerging and adjunctive approaches to surface disinfection in healthcare settings. A self-disinfecting anionic polymer surface demonstrated 99% reduction after 30 min against multiple clades, although CFU-based log reductions were not reported in that study [66]. Other exploratory interventions (i.e., compressed sodium chloride blocks, silver nanoparticles) generally yielded partial reductions/inhibition rather than consistent ≥ 5 log_10_ disinfection-level effects under the tested conditions [48,58].

### 3.8. Summary Across Disinfection Agents

Overall, across clades and test models, chlorine-based disinfectants and oxidizing chemistries (H_2_O_2_/PAA) most consistently achieved high-level reductions (often ≥5 log_10_) at practical contact times [50,56,64,65]. QAC-only products were frequently less effective and showed greater variability between clades, while QAC + alcohol formulations generally improved performance but could still demonstrate clade-dependent shortfalls [56,65]. For no-touch technologies, 254 nm UV-C produced rapid multi-log reductions under optimized dose delivery, whereas 222 nm far UV-C typically required substantially longer exposures, and ozone alone was less effective for biofilm than UV-C in direct comparison, but combined ozone + UV-C improved biofilm outcomes [54,60,61,68]. Biofilms, especially DSBs, introduce additional disinfection failure factors while transferability of fungi and regrowth may persist despite apparent CFU reductions [70].

## 4. Discussion

*C. auris* poses a distinctive infection prevention challenge because it combines efficient healthcare transmission with exceptional environmental fitness [4]. Infected patients can shed the yeast onto high-touch surfaces and shared equipment, enabling direct and indirect spread. Critically, *C. auris* can persist on dry surfaces for prolonged periods, potentially evading routine detection and facilitating recurrent recontamination after cleaning. Its capacity to form biofilms on diverse substrates and to survive despite daily cleaning and disinfection further amplifies outbreak risk once introduced into a facility [28]. Environmental hygiene is not merely supportive but central to containment bundles recommended by major public health organizations, particularly during outbreaks and in high-risk settings where environmental contamination is extensive and closely linked to patient colonization burden. Consequently, robust and validated environmental hygiene strategies integrating appropriate product selection, correct application (coverage and contact time), and protocols designed for realistic hospital conditions are central for containment of the pathogen and for interrupting transmission chains during outbreaks.

Public health agencies have issued recommendations regarding the environmental hygiene of *C. auris* in the general context of managing cases of the pathogen. The CDC recommends the use of Environmental Protection Agency (EPA)-registered hospital-grade disinfectants effective against *C. difficile* spores for the disinfection of surfaces contaminated with *C. auris*. The ECDC recommends the use of disinfectants and methods with certified antifungal activity, while the United Kingdom Health Security Agency (UKHSA, former Public Health England, PHE) advises the use of hypochlorites at concentrations of 1000 ppm [72]. In practice, products with sporicidal claims, usually chlorine-based agents and oxidizing chemistries such as hydrogen peroxide/peracetic acid, are considered effective against *C. auris* whereas QAC-only products frequently underperform and exhibit greater variability. No-touch technologies (particularly 254 nm UV-C) can provide meaningful adjunctive reductions but are highly dependent on dose delivery and deployment conditions.

Emerging data also suggest that “next generation” disinfectant candidates may arise from repurposed chemistries rather than traditional hospital biocides. From the Global Health Priority Box^®^ (240 compounds), Hydramethylnon and Flufenerim emerged as two possible candidates against *C. auris*. Hydramethylnon showed fungicidal activity, whereas Flufenerim appeared fungistatic and was further limited by evidence consistent with efflux-pump activation. Both demonstrated measurable biofilm reduction; however, these are early phase in vitro findings that still require formulation development and validation on real-world surfaces and contact times before they can be considered practical disinfectants for use in healthcare settings [73].

Overall, the current available data are in favor of sporicidal active chemistries for routine and outbreak cleaning. Chlorine agents (i.e., NaOCl, NaDCC) repeatedly achieved ≥5 log_10_ reductions across clades under practical contact times in several comparative datasets, supporting guidance that prioritizes agents with proven activity against *C. difficile* spores for *C. auris* environmental disinfection [56,64]. Oxidizing disinfectants (hydrogen peroxide, peracetic acid, and combinations) also showed robust activity, with several studies demonstrating high-level reductions across multiple clades. However, lower strength hydrogen peroxide conditions were less reliable and could be clade-dependent, underscoring the importance of formulation and use conditions [50,56,64,65].

In contrast, QAC-only formulations were the most frequently ineffective category, often failing to reach ≥5 log_10_ reduction even with extended contact times. While QAC + alcohol combinations generally improved performance and sometimes achieved ≥5 log_10_ reductions at short contact times, these benefits were not universal and clade-dependent shortfalls were reported for some formulations [56,65]. From an infection prevention and control perspective, these findings support a cautious approach to routine reliance on QAC-only “one-step” products when *C. auris* risk is present and reinforce that wipe product selection should be guided by organism-specific evidence rather than generic “hospital-grade” claims.

A critical finding of this review is the disproportion between clinical concern regarding persistence and the limited number of studies evaluating *C. auris* in biofilm state particularly DSB, which are increasingly recognized as relevant to healthcare environments [27,28]. Where DSB models were used, results challenge assumptions derived from planktonic testing. One study demonstrated a maturity-dependent decline in NaOCl efficacy in DSBs across wet/dry cycles, despite complete susceptibility of planktonic cells at the same chlorine concentrations and exposure durations. This suggests that daily disinfection may reduce surface bioburden without fully eliminating embedded reservoirs capable of persistence and reseeding [71].

Importantly, DSB-focused work also highlighted a measurement limitation: log_10_ CFU reduction alone may not adequately reflect transmission risk. In a 12-day DSB model incorporating wiping mechanics, neutralization, transfer assessment, and regrowth delay, many products that produced measurable CFU reductions still failed at clinically meaningful endpoints, namely, preventing transfer and suppressing regrowth, indicating that viable persister cells can remain transferable or rebound after treatment [70]. These findings support a shift toward endpoints that align with IPC goals (transferability and regrowth) when evaluating products intended for routine use against *C. auris* in the hospital environment.

Ultraviolet germicidal irradiation at wavelength of 254 nm can be an effective adjunctive modality, yet the observed variability reinforces that UV performance is primarily constrained by dose delivery and room geometry rather than organism biology alone [54]. For example, different emitter configurations at the same nominal delivered dose produced markedly different reductions, and distance/shadowing effects were substantial. Far-UV-C (222 nm) showed time-dependent effects and often required requiring longer exposures to achieve comparable reductions, suggesting limited feasibility to be a rapid standalone solution in many current implementations [62].

Evidence for ozone was mixed and generally indicated weaker activity against biofilm than UV-C in direct comparison, although combined ozone + UV-C strategies improved biofilm outcomes relative to ozone alone in at least one study. Overall, these data support positioning no-touch systems as adjuncts to high quality manual cleaning/disinfection, particularly for terminal cleaning or outbreak augmentation rather than replacements for chemical disinfection of high-touch surfaces [44,68].

A small but growing body of work explored adjunctive and emerging approaches such as antimicrobial/self-disinfecting surfaces, silver nanoparticles, and other exploratory materials. Reported effects were often partial (inhibition or percentage reduction) rather than consistent disinfection-level outcomes (≥5 log_10_). In addition, several studies did not report standardized CFU-based log reductions, limiting direct comparability with conventional disinfectant efficacy data [48,58,66].

Although isolates from all major clades (I–IV) were represented in the evidence base, clade representation across studies and many experiments relied on a single strain per assay. Where multi-clade comparisons were performed, strain and clade-dependent variability was documented for some products and conditions, including QAC-related formulations and certain low-strength oxidizers. This has direct implications for both product claims and facility-level decision making, as efficacy demonstrated against one isolate under idealized conditions may not reliably predict performance across circulating lineages and real-world conditions.

Several limitations should be considered when interpreting the findings. The evidence base was dominated by laboratory carrier/suspension studies with substantial heterogeneity in methods (surface types, inoculum preparation, soil load surrogates, drying conditions, wiping mechanics, neutralization procedures, and endpoints), which limited cross-study comparability and precluded meaningful meta-analysis. Biofilm evidence, particularly for DSB, remain underrepresented despite their likely relevance to environmental persistence and recurrent contamination. Many studies relied on single strains with uneven clade representation, potentially constraining generalizability to clinically relevant persistence phenotypes and circulating lineages. Outcome reporting was not fully standardized (i.e., log_10_ CFU reduction versus “no growth” or percent reduction), and relatively few studies linked environmental interventions to patient-centered outcomes such as acquisition or incidence; therefore, laboratory efficacy cannot be assumed to translate directly into transmission reduction in practice. Finally, because eligibility was restricted to English-language publications, linguistic bias may have excluded relevant evidence from non-English settings and jurisdictions.

Future research should prioritize standardized, clinically realistic test conditions (including wiping and soil load), multi-clade strain panels, routine incorporation of biofilm/DSB models, and endpoints aligned with IPC goals, particularly prevention of pathogen transfer and regrowth suppression, besides CFU reduction alone. Collectively, these steps would strengthen the bridge between “efficacy on carriers” and “reliable interruption of transmission” in busy healthcare environments.

## 5. Conclusions

Overall, the evidence from 2017 to 2025 indicates that environmental survival and surface persistence are central to *C. auris* transmission in healthcare settings, making effective environmental hygiene a core control measure. Across the reviewed studies, chlorine-based disinfectants and validated oxidizing chemistries (i.e., hydrogen peroxide/peracetic acid formulations) showed the most consistent high-level activity, whereas QAC-only products frequently underperformed and should be used with caution where *C. auris* risk exists. The available biofilm evidence, though limited, particularly for DSB, suggests that planktonic efficacy data may overestimate effectiveness in real-world conditions. Therefore, protocols and product evaluations should incorporate wiping dynamics, transfer prevention, and regrowth suppression. Strengthening the evidence base will require more standardized, clinically realistic testing and more applied studies linking environmental interventions to contamination and acquisition outcomes.

## Figures and Tables

**Table 1:** Disinfection agents tested against *Candidozyma auris*.

Agent [Abbreviations: See Footnote]	Concentration/ Wavelength	Contact Time	Logarithmic Reduction	Clade/ Strain	Biofilm	Country	Reference
NaOCl	1000 ppm	5 min, 10 min	5 min: ~2.5 log_10_ steel surface, ~1.29 log_10_ polymer surface 10 min: ~3.5 log_10_ polymer surface	Clade I and III	No	UK	[25]
	10,000 ppm	5 min	~3.5 log_10_ polymer surface				
PAA	2000 ppm	5 min	≥6 log_10_ polymer surface, ~3.5 log_10_ steel surface				
NaOCl	6500, 4000, 2000, 1000, and 500 ppm	1 min, 2 min, 4 min	Both agents ≥ 6 log_10_ (4 min at ≥1000 ppm) and >3 log_10_ (4 min at 500 to 536 ppm) >3 log_10_ at ≥4000 ppm (1 min)	Clade I	No	USA	[37]
NaDCC	4306, 1076, and 536 ppm						
Aerosolized PAA and H_2_O_2_	22% H_2_O_2_ and 4.5% PAA	21 min	≥5 log_10_ steel carriers and portable equipment	Clade I, II, III, IV	No	USA	[38]
Far Ultraviolet-C (UV-C)	222 nm	45 min and 2 h	>3 log_10_, 2 h exposure, and ≥1.2 log_10_ (range, 1.2 to 4.2 log_10_) after 2 h for all organisms at all sites.	Clade IV	No	USA	[39]
Ultraviolet-C (UV-C)	254 nm, (360° coverage 2.7 mJ/cm^2^ per second for directly exposed surfaces in 1 m distance)	20–20 min	1–2 log_10_ (significant variability between strains).	Clade I, II, III, IV	No	Austria	[40]
Chlorine	200 ppm and 500 ppm	1, 5, 30 min	>5 log_10_ 500 ppm, <3 log_10_ 200 ppm (depending on strain and contact time)	Clade I, IV	No	Turkey	[41]
CHX	4%		>5 log_10_				
CHX liquid soap	2%		>3 log_10_ Clade I, <3 log_10_ Clade IV				
Ethyl alcohol	70%		>5 log_10_				
Ultraviolet-A (UV-A)	365 nm	8 h	0.7 log_10_	Clade II	No	USA	[42]
PX-UV	200–280 nm	10, 20, 30 min 1.5 m distance	0.92 log_10_ Clade I (30 min)	Clade I, II, III, IV	No	USA	[43]
			1.38 log_10_ Clade II (30 min)				
			0.19 log_10_ Clade III (30 min)				
			1.18 log_10_ Clade IV (30 min				
Chlorine	1000 ppm	1 min	No growth (log_10_ not reported)	Clade I	No	China	[44]
Chlorine	500 ppm	30 min	No growth (log_10_ not reported)				
Ethanol	75% (*v*/*v*)	1 min	No growth (log_10_ not reported)				
Benzalkonium bromide	2000 ppm	10 min	Resistance				
UV-C light	253.7 nm	30 min	4 log_10_ (dependent on contact time and distance)				
LK/CXD bed unit ozone disinfection machine	O_3_ ≥ 300 mg/m^3^	20 min	3.57 (±0.02) log_10_				
UV-C	252.4 to 279.5 nm	5, 10, 30, and 40 s	4.81 log_10_ (≈99.998% reduction) using 267 nm and 270 nm (40 s). Effective antibiofilm activity.	Clade II	In vitro biofilm formation assay	USA	[45]
UV-C	254 nm	20 min	1.79 log_10_ reduction (98.38%)	NR	No	S. Africa	[46]
Aerosolized H_2_O_2_ (aHP)	silver-stabilized 6% H_2_O_2_	1 h	~0.29–0.36 log_10_ reductions (mean ~52% kill)				
PX-UV	200–280 nm in short pulses	5, 10, 15 min	15 and 10 min 1 m distance: no growth 5 min 1 m: 99.4% CFU reduction 15 min 2 m: no growth 10 min 2 m: 99.6% CFU reduction 5 min 2 m: 90.2% CFU reduction	NR	No	S. Africa	[47]
Compressed sodium chloride (CSC)	97.5–100% sodium chloride compressed into blocks	1 min	2 log_10_ (99% reduction with the pilot stamp method)	Clade II	No	Canada	[48]
QAC 1	0.14% BC	3 min	2.81 ± 0.64 log_10_	Clade II	No	USA	[49]
QAC 2 (towelette and 1:256 dilution)	0.125% BC	3 min towelette and 10 min 1:256 dilution	2.09 ± 0.12 log_10_ (towelette) and 2.03 ± 0.12 log_10_ (1:256 dilution)				
QAC + Alcohol	0.25% BC + 55% isopropyl alcohol	2 min	1.97 ± 0.17 log_10_				
H_2_O_2_	0.5% hydrogen peroxide	1 min	2.82 ± 0.58 log_10_				
H_2_O_2_	0.5% hydrogen peroxide	1 min	5.64 log_10_ (Cl. I), 5.20 log_10_ (Cl. II), 5.74 log_10_ (Cl III), 4.78 log_10_ (Cl. IV)	Clade I, II, III, IV	No	USA	[50]
QAC 1	BC 8.2%		1.51 log_10_ (Cl. II), <1 log_10_ (Cl. I, III, IV)				
QAC 2	BC 1.19%		1.80 log_10_ (Cl. I), 5.20 log_10_ (Cl. II), 2.18 log_10_ (Cl. III), 2.31 log_10_ (Cl. IV)				
QAC + Alcohol 1	BC 0.25% + Isopropyl alcohol 55%		5.64 log_10_ (Cl. I) 5.20 log_10_ (Cl. II), 5.74 log_10_ (Cl. III) 4.22 log_10_ (Cl. IV)				
QAC + Alcohol 2	BC 0.28% + isopropanol 17.2%		2.47 log_10_ (Cl. I), 5.20 log_10_ (Cl. II), 3.36 log_10_ (Cl. III), 4.08 log_10_ (Cl. IV)				
1-step anionic surfactant disinfectan	Dodecylbenzenesulfonic acid 0.29%		5.64 log_10_ (Cl. I), 5.20 log_10_ (l. II), 4.97 log_10_ (Cl. III), 4.78 log_10_ (Cl. IV)				
UV-C	254 nm	NR	5 log_10_ UV (range 66–110 mJ/cm^2^)	Clade I, II, III, IV	No	USA	[51]
H_2_O_2_	3%	30 s	68.6% (≈0.5 log_10_)	NR	No	S. Africa	[52]
NaDCC	500 ppm	30 s	100% (>2 log_10_ complete kill)				
Ethanol	70%	30 s	95.9% (≈1.32 log_10_)				
		60 s	100% (>2 log_10_ complete kill)				
QAC	95% BC	30 s	100% (>2 log_10_ complete kill)				
UV-C	253.7 nm	10 min	89.3% Plastic, 100% Glass, 98.9% Steel	NR	No	Poland	[53]
UV-C	254 nm	7 min	>3.86 log_10_ (99.97%) 2.4 m distance	Clade IV	No	USA	[54]
H_2_O_2_ (dry gas-vaporized)	8 g H_2_O_2_/m^3^ room space	NR	96.6–100% (3 separate experiments, one Indian isolate showed occasional survival in some replicates).	NR	No	UK	[55]
NaDCC	1000 ppm	3 min, 3 and 30 h	100% 3 min				
	10,000 ppm		Some *C. auris* (and other *Candida* spp.) were only killed at concentrations > 1000 ppm at ≥3 min contact time (tolerance compared to 1000 ppm).				
NaOCl (wipe and dilution)	0.65%	3 min	>5 log_10_ all Clades	Clade I, II, III, IV	No	USA	[56]
NaOCl (wipe)	0.63%	4 min	>5 log_10_ all Clades				
NaDCC	4306 ppm	4 min	>5 log_10_ all Clades				
PAA, H_2_O_2_	1200 ppm, H_2_O_2_ < 1%, acetic acid	3 min	>5 log_10_ all Clades				
H_2_O_2_	1.4% (sporicidal activity due to generation of low concentrations of PAA during use)	3 min	>5 log_10_ all Clades				
H_2_O_2_	0.5%	5 min	>5 log_10_ all Clades				
H_2_O_2_	0.07%	10 min	4.02 log_10_ Clade II, 1.48 log_10_ Clade I, not effective on Clade III and IV				
H_2_O_2_	4%	1 min	>5 log_10_ all Clades				
QAC + Alcohol 1	BC + Isopropyl alcohol 55%	2 min	>5 log_10_ all Clades				
QAC + Alcohol 2	BC + Isopropanol 17.2%	3 min	Spray achieved >5 log_10_ on Clade II, for the rest Clades <5 log_10_				
Phenolic acid	Orthophenylphenol 3.4%, ortho benzyl para chlorophenol 3.0%	10 min	No >5 log_10_ reduction in any of the test strains.				
QAC	BC	3–10 min	None of the quaternary ammonium–based products achieved a >5 log_10_ reduction				
Biofilm-disrupting agents (BDs).	100% and 20%	Planktonic: 1, 5, 10, 30, 60 min	Planktonic: 100% (≈7-log_10_) 1 min 20% dilution ≈ 6 log_10_ 10 min	Clade II and IV	In vitro biofilm formation assay	USA	[57]
		Biofilm: 24 h	Biofilm: ≈ ≥7 log_10_ reduction (~99.99999%) for 100% concentration				
AgNPs	0.008 to 1.15 ppm and 0.017–2.3 ppm (polystyrene)	24 h	50% inhibition	Clade I	In vitro biofilm formation assay	USA	[58]
	0.017–2.3 ppm (Elastic bandage fibers)		>80% inhibition				
H_2_O_2_	4.04%, <10% acetic acid	1 min	>6.0 log_10_ Clade II, ≥5.1 log_10_ Clade IV	Clade II, IV	No	USA	[59]
NaOCl	0.65%		>6.1 log_10_ Clade II, ≥6.6 log_10_ Clade IV				
PAA + H_2_O_2_	0.13% PAA, 0.63% H_2_O_2_		>5.1 log_10_ Clade II, ≥5.1 log_10_ Clade IV				
Accelerated H_2_O_2_ + alcohol	H_2_O_2_ >0.1–<1%, benzyl alcohol 1–5%		>5.4 log_10_ Clade II, ≥5.1 log_10_ Clade IV				
Far Ultraviolet-C (UV-C)	222 nm	4 h, 12 h	>2 log_10_ CFU 4 h >3 log_10 CFU_ 12 h	Clade II	No	USA	[60]
Far Ultraviolet-C (UV-C)	222 nm	45 min	<3 log_10_ Not effective	Clade II, III	No	USA	[61]
UV-C	254 nm (configuration: 3 emitters in a linear/row layout, rotating 5°)	12 min	>6.0 log_10_ (575 mJ/cm^2^ delivery to 2.7 m)	Clade II	No	USA	[62]
	254 nm (configuration: 3 emitters triangular; rotate 360°)	18 min	1.66 log_10_ (575 mJ/cm^2^ delivery to 2.7 m)				
Detergent	Not reported	NR	3.69 log_10_ (Microfiber and cotton mop)	Clade IV	No	USA	[63]
QAC	BC		3.26 log_10_ (Microfiber Mop) 3.69 log_10_ (Cotton mop)				
NaOCl	0.25%		>3.85 log_10_ ND (Microfiber Mop) 3.69 log_10_ (Cotton mop)				
Water			3.51 log_10_ (Microfiber Mop) 2.45 log_10_ (Cotton Mop)				
UV-C	254 nm	10 min	>3.85 log_10_ ND (91 cm distance)				
NaOCl	0.65%	1 min	>5 log_10_	Clade II	No	USA	[64]
NaOCl	0.39%	1 min	>5 log_10_				
NaOCl	0.825% (diluted)	1 min	>5 log_10_				
PAA, H_2_O_2_	1200 ppm, H_2_O_2_ < 1%	3 min	>5 log_10_				
H_2_O_2_	1.4%	1 min	>5 log_10_				
H_2_O_2_	0.5%	10 min	>5 log_10_				
Acetic acid	>5% (pH 2.0)	3 min	≈2–3 log_10_				
Ethyl alcohol	29.4%	30 s	≈2–3 log_10_				
QAC 1	BC	10 min	≈1–2 log_10_				
QAC 2	BC	10 min	≈1–2 log_10_				
H_2_O_2_	0.5% H_2_O_2_	10 min	≥5.32 log_10_	Clade IV	No	USA	[65]
H_2_O_2_	0.5% H_2_O_2_	1 min	≥5.48 log_10_				
H_2_O_2_	1.4% H_2_O_2_	3 min	≥5.48 log_10_				
QAC 1	0.084%	10 min	1.82 log_10_				
QAC 2	10.9%	10 min	0.56 log_10_				
QAC 3	21.7%	10 min	0.56 log_10_				
QAC 4	2%	10 min	0.25 log_10_				
QAC + Alcohol 1	0.61% BC + 28.7% Isopropanol + 27.3% ethanol	1 min	≥5.29 log_10_				
QAC + Alcohol 2	0.5% BC + 55% isopropanol	2 min	≥5.29 log_10_				
Anionic pentablock polymer with 52 mol% midblock sulfonation	Anionic block polymer that generates layer that is acidic (pH < 1) upon hydration	30 min	99% reduction	Clade I, III, IV	No	USA	[66]
Alcohol	35% (25 g ethanol (94%), 35 g propan-1-ol)	30 s	4 log_10_	Clade II	No	Germany	[67]
QAC	0.25%	1 min	4 log_10_				
UV-C + O_3_	254 nm + 50 ppm	20, 40, 60 min	≈4 log_10_ BF and 8 log_10_ PC	Clade I	In vitro biofilm formation assay	UK	[68]
UV-C	254 nm		≈7.2 log_10_ BF and 8 log_10_ PC 40 min				
O_3_	50 ppm (1000–3000 ppm·min)		≈3.3 log_10_ BF and 8 log_10_ PC 40 min				
Alcohol	100%	1 min	>5 log_10_ (no growth) PC and BF	Clade I, II, III	In vitro biofilm formation assay	Austria	[69]
QAC	100%	1 min	>5 log_10_ (no growth) PC and BF				
ALD + QAC	0.5%	15 min	>5 log_10_ (no growth) PC and BF				
H_2_O_2_	3.4% (*v*/*v*)	5 min	>5 log_10_ (no growth) PC Incomplete, strain-dependent for BF				
H_2_O_2_	4.25% (*v*/*v*)	5 min	>5 log_10_ (no growth) PC <3 log_10_ for NCPF8971 variability reported for BF				
PP	3%	30 min	>5 log_10_ (no growth) PC and BF				
PAA	3500 ppm	2 min	>7 log_10_	Clade II	In vitro biofilm formation assay	UK	[70]
	250 ppm		0.84 log_10_				
NaOCl	1000 ppm		>7 log_10_				
NaDCC	1000 ppm		>7 log_10_				
	10,000 ppm		≈4 log_10_				
QAC	BC < 0.5% (<5000 ppm)		4 log_10_				
ClO_2_	300 ppm		<2.5 log_10_				
	1000 ppm						
NaOCl	500 ppm–1000 ppm	1 min	≥6 log_10_ PC, 4.5 → 1.2 log_10_ from cycle 1 to cycle 3 BF	Clade I, III	In vitro biofilm formation assay	UK	[71]
	1000 ppm	5 min	≥6 log_10_ PC, 6.7 → 3.0 log_10_ from cycle 1 to cycle 3 BF				

AgNPs: Silver nanoparticles, aHP: Aerosolized hydrogen peroxide, ALD: Aldehyde, BC: Benzalkonium chloride, BF: Biofilm, CHX: Chlorhexidine, ClO_2_: Chlorine dioxide, H_2_O_2_: Hydrogen peroxide, NaDCC: Sodium dichloroisocyanurate, NaOCl: Sodium hypochlorite, ND: Not detected, NR: Not reported, O_3_: Gaseous ozone, PAA: Peracetic acid, PC: Planktonic cells, PP: Potassium peroxymonosulfate, QAC: Quaternary Ammonium Compound, PX-UV: Pulsed-xenon ultraviolet-C light, UV-A: Ultraviolet-A, UV-C: Ultraviolet-C, UVGI: Ultraviolet germicidal irradiation.

## Data Availability

The original contributions presented in this study are included in the article. Further inquiries can be directed at the corresponding authors.

## Abbreviations

The following abbreviations are used in this manuscript:

AMR	Antimicrobial Resistance
CDC	Centers for Disease Control and Prevention
DSB	Dry-Surface Biofilm
ECDC	European Centre for Disease Prevention and Control
HAI	Healthcare-Associated Infections
HEH	Healthcare Environmental Hygiene
IPC	Infection Prevention and Control
MDRO	Multidrug-Resistant Organism
QAC	Quaternary Ammonium Compounds
UKHSA	United Kingdom Health Security Agency
